# Supplementary material for: Retinal organoids mirror CRISPR-Cas9 gene editing efficiency observed in vivo
Source: Mol Ther Methods Clin Dev. 2025 Nov 10;33(4):101627. doi: 10.1016/j.omtm.2025.101627 (PMC12686912; doi:10.1016/j.omtm.2025.101627)
Supplement: Document S1. Figures S1–S6 and Tables S1–S6 [file mmc1.pdf]

OMTM, Volume *33*

Supplemental information

Retinal organoids mirror CRISPR-Cas9 gene editing efficiency observed *in vivo*

Juliette Pulman, Hugo Malki, Paul Oudin, Ecem Aydin, Sophie Tran, Laura Visticot, Camille Robert, Anne De Cian, Marie As, Olivier Goureau, Jean-Paul Concordet, and Deniz Dalkara

Supplemental results:

(See additional videos)

Video S1: Timelapse imaging of non-transfected HEK293T cells.

Non-transfected HEK293T cells were imaged over a 42-hour period. Cell growth and division are observed throughout the experiment. Live images were taken every 15min.

Video S2: Timelapse imaging of CRISPR/Cas9 transfection in HEK293T cells.

Cas9-GFP RNP mixed with lipofectamine complexes (in green) interact with HEK293T cells over a 42-hour period after transfection. Capture of an RNP aggregate by a cell is highlighted in two zoomed-in sections. Live images were taken every 15min.

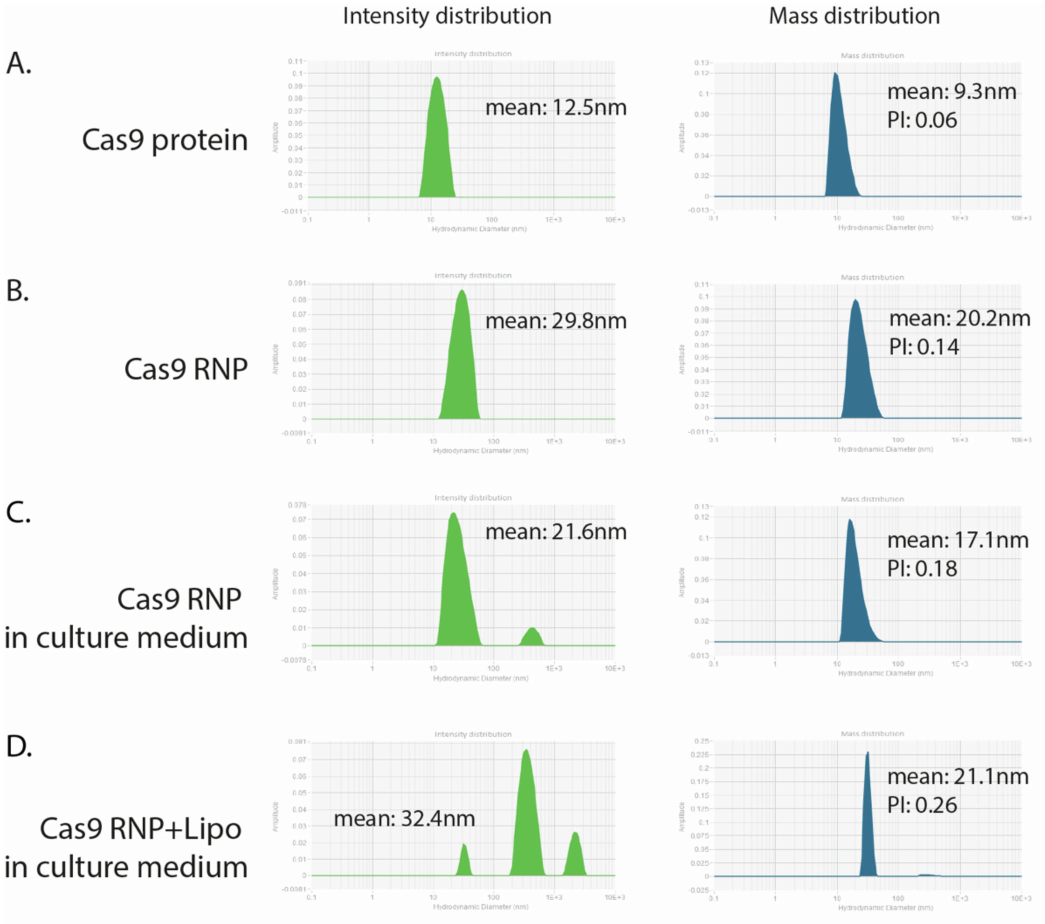

Figure S1. Hydrodynamic size of Cas9 RNP and Cas9 RNP complexed with lipofectamine 2000 in culture medium.

Size distribution by intensity and mass of Cas9 protein and its sgRNA: (A) of the Cas9 protein alone (B) of the Cas9 protein mixed with the sgRNA (C) of the Cas9 protein mixed with the sgRNA and incorporated in the culture medium (D) of the Cas9 protein mixed with the sgRNA and with Lipofectamine 2000 and incorporated in the culture medium.

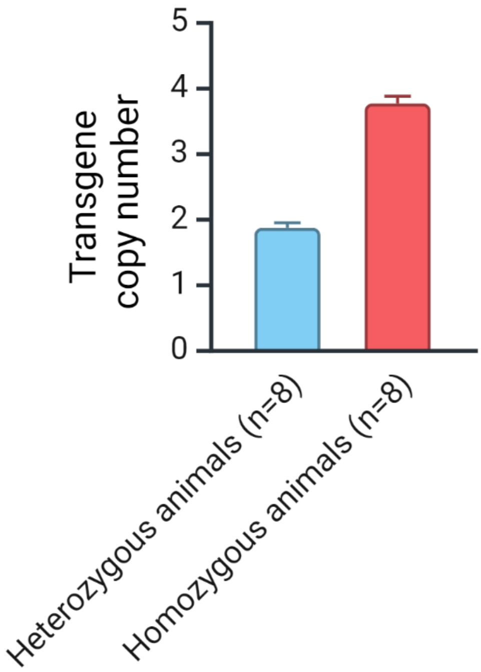

Figure S2. Transgene copy number of RHO in the RHO.P347S mouse line quantified using Droplet Digital PCR (ddPCR)

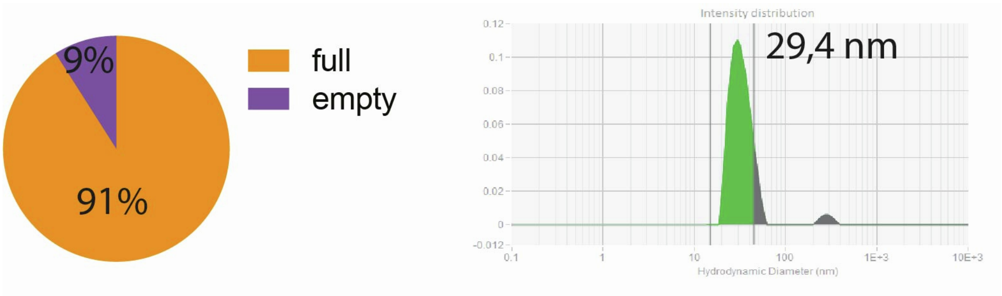

Figure S3. DLS characterization of the SpCas9 AAV construct. Left panel: pie chart showing the percentage of full (orange) vs empty (purple) AAV capsid. Right panel: hydrodynamic size distribution by intensity of AAV:SpCas9. Mean capsid diameter (nm).

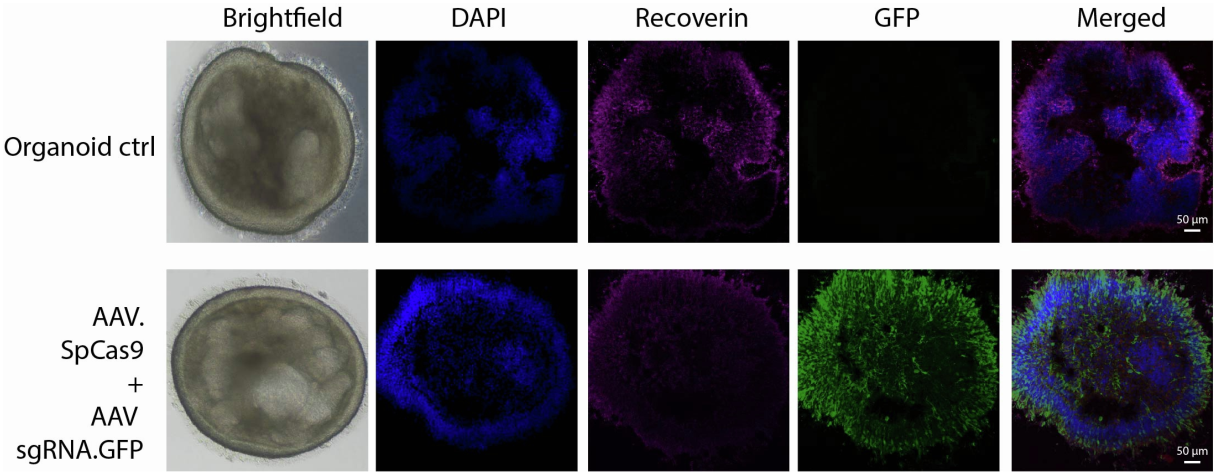

Figure S4. Immunostaining of a full organoid. Cryosection of an organoid 21 days after dual-AAV CRISPR transduction, stained with GFP and Recoverin, a photoreceptor marker.

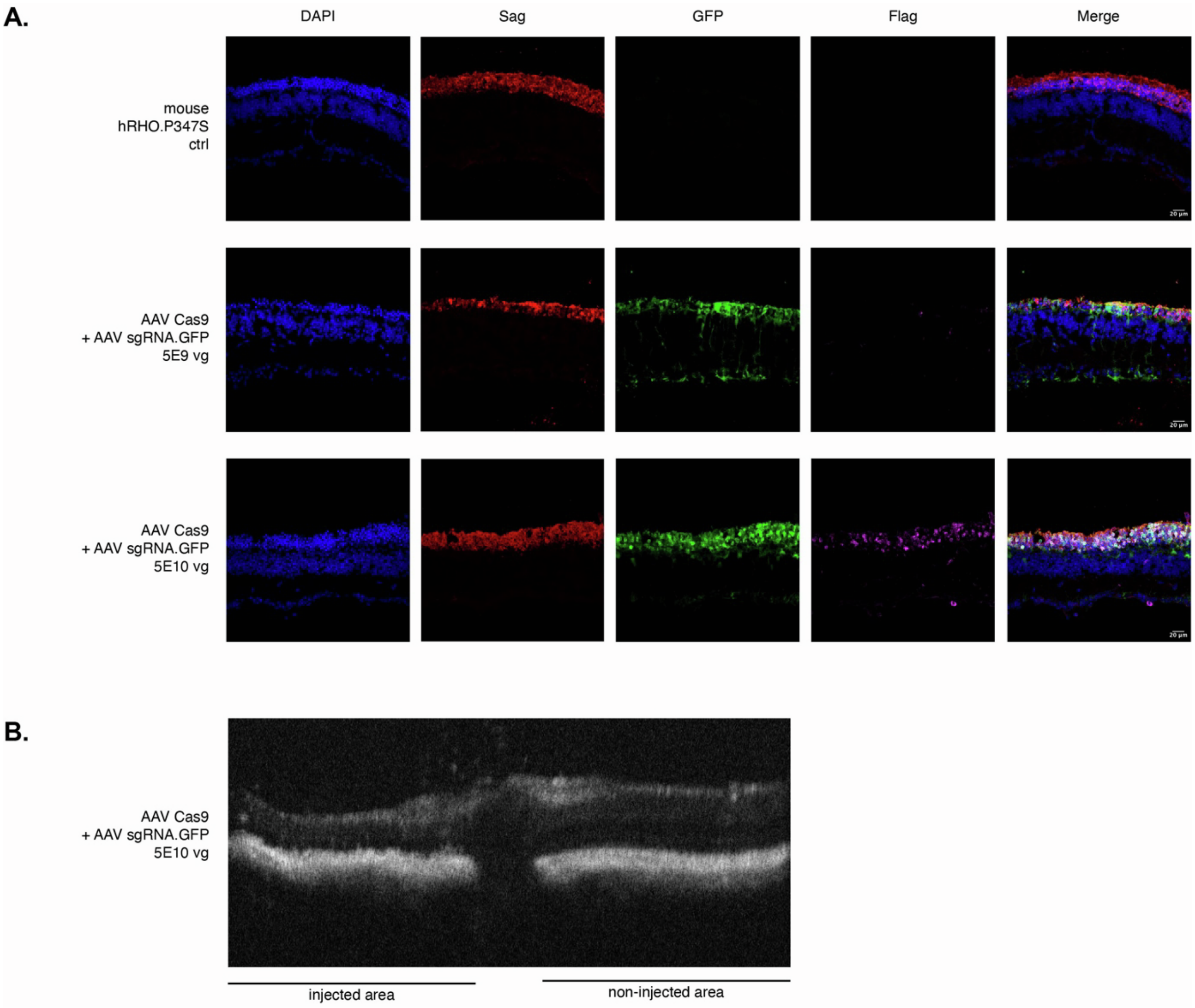

Figure S5. A. Immunostaining of SpCas9-Flag and GFP. Cryosections of neural retina of mouse hRHO.P23H stained for nuclei (DAPI, blue), photoreceptors (Sag, red), GFP (green) and SpCas9 (Flag, magenta) 3 weeks after sub-retinal injection of a total dose of 5E9 vg or 5E10 vg of the double AAV strategy. B. OCT images 3 weeks post-injection of CRISPR/Cas9 AAV at 5E10 vg in h*RHO*.p347S mouse.

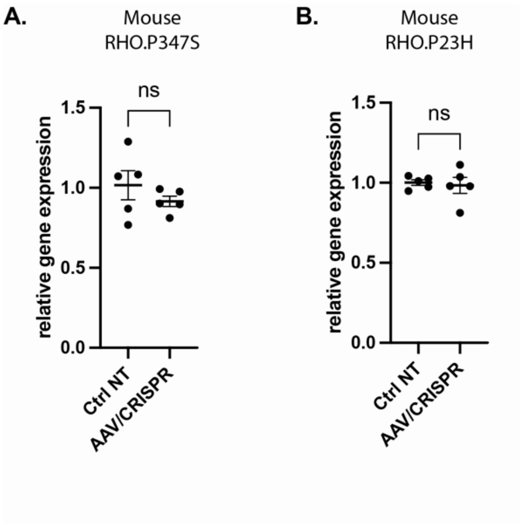

Figure S6. Relative expression of *hRHO* in the photoreceptors (A) in the P347S mouse mode (B) in the P23H mouse model. 21 days after-subretinal injection of AAV.SpCas9 and AAV.sgRNA.. Results of qPCR after 2^(-ddCT) analysis using *Pde6b* as a reference gene. Mean ± SEM.

Table S1. sgRNA sequences used in this study

sgRNA name	Position on *RHO*	Spacer (5'-3')	PAM (5'-3')
**sgRNA 1**	Exon 1	GATCAGCAGAAACATGTAGG	CGG
**sgRNA 2**	Exon 1	TTGAGCAGGATGTAGTTGAG	AGG
**sgRNA 3**	Exon 1	AGAGCGTGAGGAAGTTGATG	GGG
**sgRNA 4**	Exon 1	AGCCACCTAGGACCATGAAG	AGG
**sgRNA 5**	Exon 1	AGTACTGTGGGTACTCGAAG	GGG
**sgRNA 6**	Exon 2	TGGCATGGTTCTCCCCGAAG	CGG
**sgRNA 7**	Exon 2	CAGGACCACCAAGGACCACA	GGG
**sgRNA 8**	Exon 2	TTGCAGGTGAAATTGCCCTG	TGG
**sgRNA 9**	Exon 3	CTACTACACGCTCAAGCCGG	AGG

Table S2. Primers used to amplify the targeted regions of each gene for Sanger sequencing and TIDE analysis

Primer name	Experiment	Sequence (5'-3')
Sanger-sgRNA1-Fw	TIDE analysis	GGG GTC AGA ACC CAG AGT CA
Sanger-sgRNA1-Rev	TIDE analysis	TGG CAA AGA AGC CCT CCA AA
Sanger-sgRNA2-Fw	TIDE analysis	GAA TGG CAC AGA AGG CCC TAA
Sanger-sgRNA2-Rev	TIDE analysis	CAT TGA CAG GAC AGG AGA AGG G
Sanger-sgRNA3-Fw	TIDE analysis	ATT CTT GGG TGG GAG CAG C
Sanger-sgRNA3-Rev	TIDE analysis	ACC CAC ACC CGG CTC ATA C
Sanger-sgRNA4-Fw	TIDE analysis	ATG GCA CAG AAG GCC CTA AC
Sanger-sgRNA4-Rev	TIDE analysis	TGA CAG GAC AGG AGA AGG GA
Sanger-sgRNA5-Fw	TIDE analysis	TCA GCC AGG AGC TTA GGA GG
Sanger-sgRNA5-Rev	TIDE analysis	TGC AGA GAG GTG TAG AGG GT
Sanger-sgRNA6-Fw	TIDE analysis	GGT TGC CTT CCT AGC TAC CC
Sanger-sgRNA6-Rev	TIDE analysis	TAC ACC CCT ACC CTG AGT GG
Sanger-sgRNA7/8-Fw	TIDE analysis	TTT CTT TGC CCA GCT CTC CT
Sanger-sgRNA7/8-Rev	TIDE analysis	TGA GTC CTG ACT GGA GGA CC
Sanger-sgRNA9-Fw	TIDE analysis	GCA TCT GCA TCC CCA TCT GA
Sanger-sgRNA9-Rev	TIDE analysis	GTC CAG ACC ATG GCT CCT C

Table S3. Primers used to amplify the targeted regions of each gene for NGS sequencing

Primer name	Experiment	Sequence (5'-3')
NGS-sgRNA7-Fw	NGS-CRISPResso2	TGC TCA GTG CCA TTA CCT GGA
NGS-sgRNA7-Rev	NGS-CRISPResso2	GAG TGC ACC CTC CTT AGG C
NGS-sgTS2-Fw	NGS-CRISPResso2	TGG GCT TCC CCA TCA ACT TC
NGS-sgTS2-Rev	NGS-CRISPResso2	TGG CAA AGA AGC CCT CCA AA
NGS-sgRNA1-Fw	NGS-CRISPResso2	CCC ACA GTA CTA CCTGGC TG
NGS-sgRNA1-Rev	NGS-CRISPResso2	GGG CCC GAA GAC GAA GTA TC

Table S4. Primers used for the RT-qPCR

Primer name	Experiment	Sequence (5'-3')
qPCR-sgRNA7-Fw	sgRNA expression	CCA AGG ACC ACA GTT TTA GAG C
qPCR-sgRNA7-Fw	sgRNA expression	CGG TGC CAC TTT TTC AAG TT
qPCR-SpCas9-Fw	Cas9 expression	AAC AGC CGC GAG AGA ATG AA
qPCR-SpCas9-Rev	Cas9 expression	CAC GGG GTG TTC TTT CAG GA
qPCR-hRHO-Fw	hRHO expression	TCA TGG TCC TAG GTG GCT TC
qPCR-hRHO-Fw	hRHO expression	GGA AGT TGC TCA TGG GCT TA
qPCR-Pde6b-Fw	Ctrl for Rho expression	TCC GGG CCT ATC TAA ACT GC
qPCR-Pde6b-Rev	Ctrl for Rho expression	AGA AGA CAA TTT CCC GGC CAT

Table S5. Primary antibodies used for immunostaining

Antigen	Dilution	Source
**DAPI**	1/2000	Thermo A31573
**Recoverin**	1/3000	Millipore MAB 5585
**hCAR**	1/10.000	Homemade. Already published in [1]
**RHO**	1/500	Merck MABN15
**Peripherin**	1/250	Proteintech 18109-1-AP
**SpCas9**	1/1000	Genscript A01935-40
**GFP**	1/500	Thermo A11122
**Sag**	1/2000	Thermo PA1-731

Table S6. Primers used for the transgene copy number quantification

**Probe hRHO-P347S-HEX**	Transgene CNV	HEX/ CCT CTC AGA /ZEN/ CCC TCG CAG C
**Probe actin-FAM**	Transgene CNV	FAM/ AGG CAG CCA /ZEN/ GGG CTG GC
**hRHO-CNV-Fw**	Transgene CNV	GGA GGA ATG AAT GGG AAG GG
**hRHO-CNV-Rev**	Transgene CNV	CCA GGG AGG GAA AAA CAA CT
**Actin-CNV-Fw**	Transgene CNV	TAT GAA GGC TTT GGT CTC CC
**Actin-CNV-Rev**	Transgene CNV	CAC AGA GCC ACA AGC TGT TT

Reference

1. Garita-Hernandez M, Lampič M, Chaffiol A, et al. Restoration of visual function by transplantation of optogenetically engineered photoreceptors. *Nature Communications*. 2019;10(1). doi:10.1038/s41467-019-12330-2
